# Diseases of the Heart and Vascular System. II

**Published:** 1894-09-15

**Authors:** 


### Progress in Medicine and Surgery.

### DISEASES OF THE HEART AND VASCULAR SYSTEM.—II.

**The Influence of the Arterioles in Relation to Various Pathological Conditions** is discussed by Sir George Johnson, who refers to Dr. Rose Bradford's and Dr. Percy Dean's[5] demonstration of the existence of pulmonary vaso-motor nerves, and that the pulmonary circulation is comparatively independent of the systemic. He himself shows that the immediate cause of death from apnœa is the arrest of the pulmonary circulation. That the arrest of the circulation through the lungs is caused by contraction of the pulmonary arterioles appears to be proved by the influence of agents which are known to paralyse those vessels, viz., nitrate of amyl, atropine, and an excessive dose of curara, the result beingthat deprivation of air is unattended by distension of the right cavities of the heart and other evidence of obstructed pulmonary circulation, the life of the animal is prolonged several minutes, and death ultimately results from the toxic action of deoxidised blood upon the cardiac and nervous tissues. The resistance offered by the blood capillaries to the circulation is shown by Harry Campbell[6] to be almost nil, the smaller arteries bearing the whole force of the arterial tension. If the vascular system be regarded from a comprehensive standpoint, it will be seen that it has but one end in view, viz., the maintenance of a proper capillary circulation. This implies continuousness—*i.e.*, non-intermission of flow; (*b*) a certain average tension, which, above all, shall not be unduly high; and (*c*) the capacity of being modified in response to the fluctuating demands of the tissues.

**The Pathology of Œdema from Passive Congestion.**—Lazarus-Barlow[7] considers that the view which explains the œdema accompanying passive congestion upon purely mechanical principles is not borne out by facts, for upon this view the exudation from the blood vessels should be increased in amount synchronously with the increase of pressure, whereas no such exudation is found to take place during an hour after the pressure in the veins has been raised experimentally. The varieties of anæmia examined by him were (1) complete anæmia, lasting three hours; (2) hæmostasis, or cutting off of the limb, with its contained blood and lymph, from the rest of the circulation for one hour, by means of a ligature; (3) complete anæmia combined with stimulation of the sciatic nerve, and persistence, *in situ*, of the products of muscle metabolism, the whole lasting one hour. It was found that in each of these conditions œdema occurred. The conclusions he arrived at was that starvation of the tissues plays an important part in the occurrence of œdema; the amount of œdema is greater in the cases of venous congestion, and varies directly with the duration of the action of venous blood, and lastly that the products of metabolism play a part more important than starvation. The greatest amount of œdema was obtained with venous obstruction after anæmia and stimulation of the sciatic nerve.

### Diagnosis of Cardiac Diseases.

**The Effect of Pressure in Modifying Murmurs** is discussed by Professor Sidney Ringer and Dr. Mear.[5] They remind us that generally pressure of the stethoscope on the chest wall markedly diminishes the intensity of cardiac bruits, and raises the pitch. Occasionally a pulmonary hæmic bruit is increased in intensity by pressure.

They point out that in the majority of cases of hæmic murmur which is weakened by pressure the production of the murmur cannot be supposed to be modified, since this change in character may be obtained equally well, or even more readily, at a distance from the point of production than immediately over this point; the modification, therefore, has to do solely with conduction. They also note the effects on murmurs of slight changes in posture.

**Bruits and Murmurs.**—Aortic incompetence without a diastolic bruit is now a recognised possibility. Professor Leyden[9] showed a heart in which these peculiar conditions had existed during life, stating that it would appear that the presence of aortic incompetence is not necessarily associated with an abnormal bruit on auscultation, and he himself had had two such cases. Professor Gerhardt, in the discussion which followed, remarked that the presence of a certain degree of contraction of the calibre of the passages is essential for the production of cardiac murmurs, which vary in intensity according to the degree of contraction and the velocity of the blood current. He was fully aware that no diastolic sound may be heard in cases of incipient aortic incompetence, but the appearance of the bruit in question is seldom long delayed. Moreover, there may be an absence of diastolic murmur in cases in which the aortic incompetence is complicated by other valvular lesions, such, for example, as mitral incompetence, when the blood does not travel with sufficient rapidity to give rise to the production of a diastolic bruit.

Dr. Frank Jackson[10] refers to a source of error in the diagnosis of heart disease, one that he believes is almost altogether overlooked—viz., the fact that some murmurs are heard only by the unaided ear and are inaudible to the stethoscope, while some are inaudible to the ear and are heard with great distinctness by the stethoscope. This curious characteristic of murmurs may be noticed in any form of valvular disease, but it is most common in connection with the murmur of aortic regurgitation. Though the fact is unmistakable, he can afford no explanation of these remarkable phenomena.

**Bruits without Valvular Disease.**—Jackson states that it is not uncommon to discover cardiac murmurs in patients who have never experienced the slightest symptom of derangement of the heart's action, and in many of whom no evidence of hypertrophy or other compensation can be found. A little leakage through a valve by no means destroys the efficiency of the valve, and, in the absence of evidence of progressive endocarditis, such cases should not be regarded as true heart disease.

**Cardiac Disease without Bruits** presents cases of far greater difficulty for diagnosis. This applies especially to disease of the aortic valves, or of the aorta just above the valves. Jackson draws particular attention to the class of cases in which there is either atheroma or thickening of the aortic valve with atheroma of the aorta, or there is atheroma of the aorta in the neighbourhood of the orifices of the coronary arteries without involvement of the aortic valves. Such cases generally occur in overworked men of above middle-age, and among the upper classes. These men complain for a considerable time of "dyspepsia"; then they have painful sensations about the heart, then palpitation of the heart, and finally dyspnœa on exertion. They are liable to sudden death, usually with symptoms of *angina pectoris*. It seems to him that the most significant signs of such a condition are enlargement of the heart and an absence of the aortic valve sound, or a feeble aortic valve sound, though both these signs may be absent.

**Reduplication of the First Sound.**—In discussing this peculiarity of the first sound of the heart, O. J. Kauffmann "excludes those cases known as the *bruit de galop*, in which the first sound is simply doubled, referring only to those in which a very short but appreciable interval separates the two component sounds, although the two phenomena are probably nearly related to one another as regards their pathology. The first part of the double sound appears as a prefix to the first sound, and never coincides in time with the apex impulse. The writer bases his observations on an analysis of thirty-four cases in his own experience. The greatest number of instances occurred in Bright's disease, and next in frequency were those associated with dilatation of the heart, especially of the left ventricle. In the great majority the reduplication was evanescent, and the fact that only one case ended fatally precludes the usually accepted view that the phenomenon is of bad augury. In no single case did the reduplication occur in chronic valvular disease, even in the latest stages. The writer, therefore, excludes valvular disease as a cause of the trouble, and there was ample evidence that it bore no relation to high or low arterial tension.

[5] Proc. Ryl. Soc., vol. xlv., p. 369. [6] Lanc., March 10, 1894. [6] Commr. to Royal Soc., Dec., 1893; B. M. J. supp., April 21 1894. [8] Lancet, Feb. 10, 1894. [9] Med. Week, Feb. 9, 1894. [10] New York Med. Journ., April 28, 1894.

